# Supplementary material for: Evaluation of Linkers’ Influence on Peptide-Based Piezoelectric Biosensors’ Sensitivity to Aldehydes in the Gas Phase
Source: Int J Mol Sci. 2023 Jun 25;24(13):10610. doi: 10.3390/ijms241310610 (PMC10342081; doi:10.3390/ijms241310610)
Supplement: Supplementary file 1 [file ijms-24-10610-s001.zip › ijms-2409431-supplementary.pdf]

GSGSGS	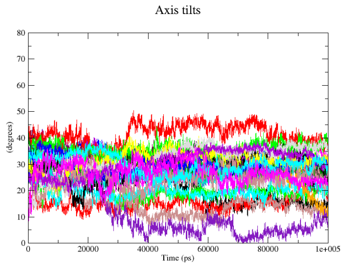	GGGGS	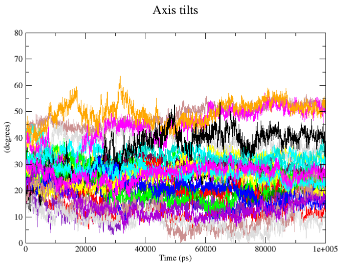
PPPP	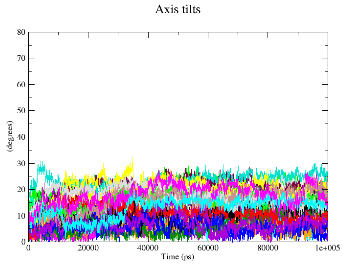	PPP	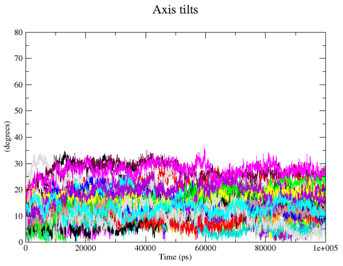
4*βA	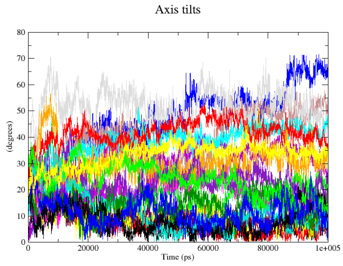	GABA	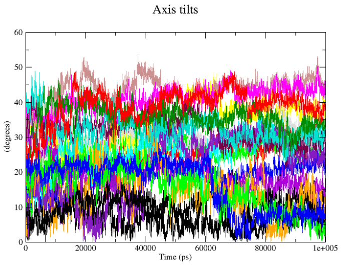
GABA*2	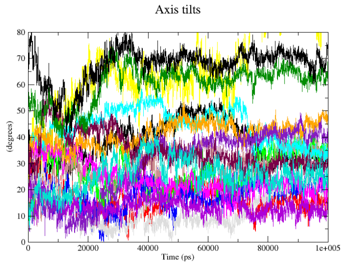	6-Ahx	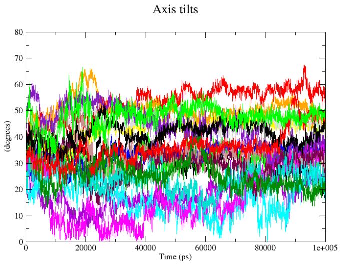

Figure S1. The charts represent the change in helix axis tilt of peptides during a 100 ns molecular dynamics simulation for peptides with different linkers.

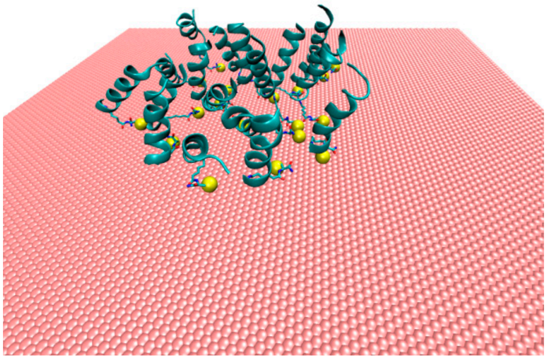 Structure of the receptor with 6*Ahx linker	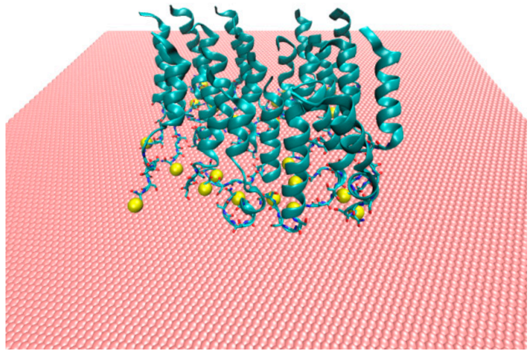 Structure of the receptor with GSGSGS linker

Figure S2. Representative structures of receptors from both groups (proteogenic and non-proteogenic).

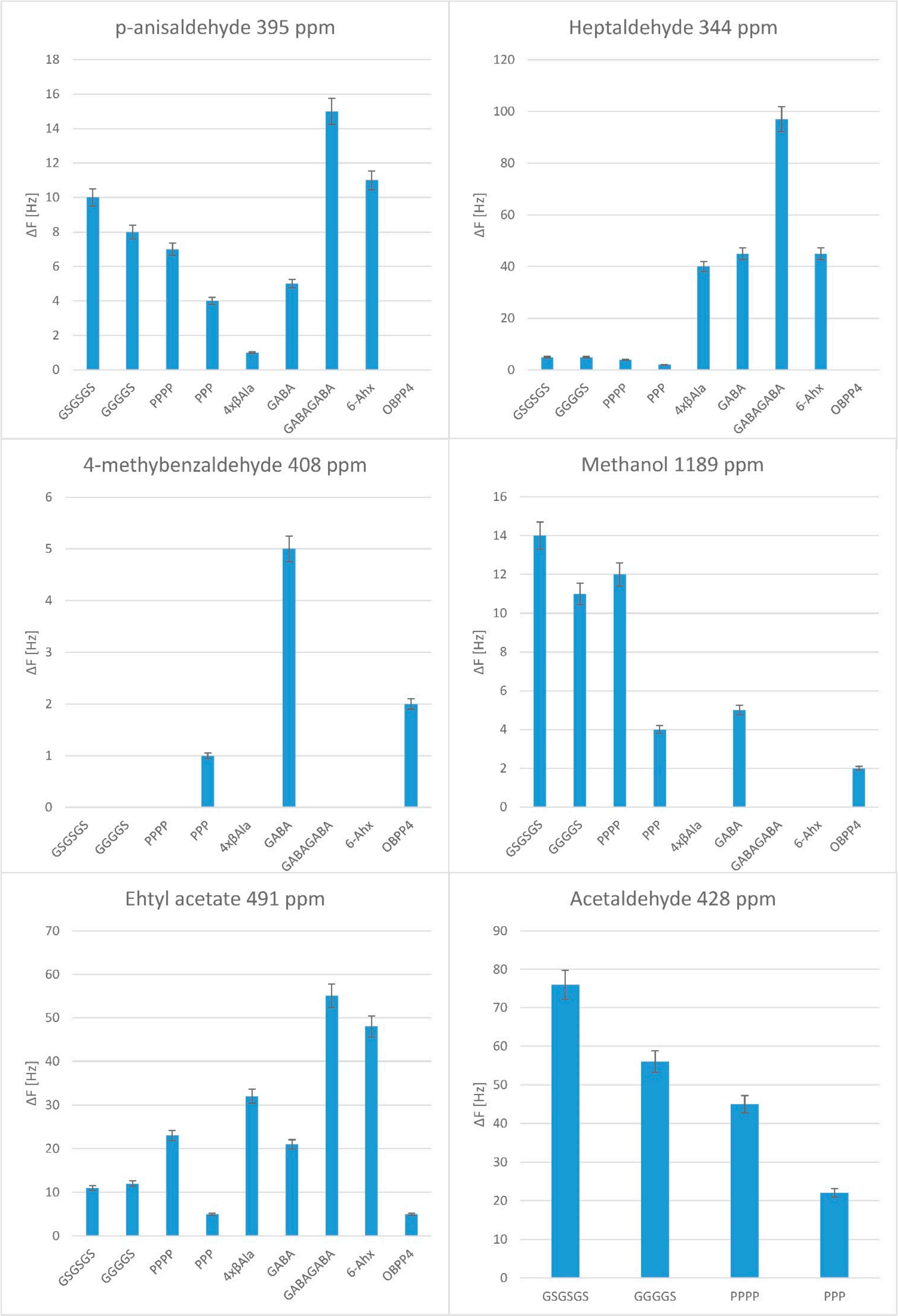

Figure S3. The changes of resonance frequencies (ΔF) in the presence of different gaseous compounds.
